# Leakage after Surgery for Rectum Cancer: Inconsistency in Reporting to the Danish Colorectal Cancer Group

**DOI:** 10.1155/2015/376540

**Published:** 2015-11-09

**Authors:** L. Borly, M. B. Ellebæk, N. Qvist

**Affiliations:** Surgical Department A, Odense University Hospital, Denmark

## Abstract

*Purpose*. Anastomotic leakage accounts for up to 1/3 of all fatalities after rectal cancer surgery. Evidence suggests that anastomotic leakage has a negative prognostic impact on local cancer recurrence and long-term cancer specific survival. The reported leakage rate in 2011 in Denmark varied from 7 to 45 percent. The objective was to clarify if the reporting of anastomotic leakage to the Danish Colorectal Cancer Group was rigorous and unequivocal. *Methods*. An Internet-based questionnaire was e-mailed to all Danish surgical departments, who reported to Danish Colorectal Cancer Group (DCCG) in 2011. There were 23 questions. Four core questions were whether pelvic collection, fecal appearance in a pelvic drain, rectovaginal fistula, and “watchfull” waiting patients were reported as anastomotic leakage. *Results*. Fourteen out of 17 departments, who in 2011 according to DDCG performed rectal cancer surgery, answered the questionnaire. This gave a response rate of 82%. In three of four core questions there was disagreement in what should be reported as anastomotic leakage. *Conclusion*. The reporting of anastomotic leakage to the Danish Colorectal Cancer Group was not rigorous and unequivocal. The reported anastomotic leakage rate in Danish Colorectal Cancer Group should be interpreted with caution.

## 1. Introduction

A unique international accepted definition of anastomotic leakage (AL) is paramount to gather knowledge about the true incidence of AL and to perform valid comparison between different departments, regions, or countries. Furthermore it is important for the study of risk factors and the consequences of AL on local cancer recurrence and long-term cancer specific survival [1, 2].

Another problem is the different clinical presentation of AL, which includes peroperative demonstrated leakage, air, or intestinal content in drain, pelvic sepsis, or leakage demonstrated by a CT-scan, suture line dehiscence demonstrated by endoscopy, and overt peritonitis.

In Denmark all departments performing colorectal cancer surgery are obliged to report their results to the Danish Colorectal Cancer Group (DCCG) [3]. The DCCG database is a prospective, nationwide database with a patient completeness rate of 99%. One of the quality indicators in the DCCG yearly report is the individual department AL frequency, which must be no more than 10%.

In 2011 seventeen Danish surgical departments reported their results to the DCCG database. Out of 382 patients with rectum cancer who underwent a colorectal or coloanal anastomosis, 51 patients or 13.35% were reported having an AL. The reported department frequency varied from 7 to 45 percent [4]. The Danish national guidelines did not include any strict definitions on anastomotic leakage and there might be a risk of inconsistent reporting of AL.

The aim of the present study was by a structured questionnaire to clarify whether the reporting of AL to the DCCG database could be considered as rigorous and unequivocal.

## 2. Material and Methods

In March 2013 a self-administrated Internet-based questionnaire was e-mailed to all Danish surgical departments who reported to the DCCG database in 2011. The departments received a reminder after 2 months followed by information on the project by phone in order to maximize response rate.

There were 23 questions, which were a mixture of open format, closed format, and leading questions. The different formats were used when appropriate. An online survey service (Survey Monkey) was used.

## 3. Results

Fourteen out of a total of 17 departments answered the questionnaire. This gave a response rate of 82%. One department had not performed surgery for rectum cancer and was excluded. Thus, thirteen responders were available for the per protocol analysis. None of the responders reported having made significant changes in their definition or reporting of AL to the database within the last two years. The 13 departments who answered the questionnaire represented 94% of all rectum resections and 93% of all AL in Denmark in 2011.

Eight out of thirteen departments answered yes to the existence of a department approved guideline for diagnosing AL. Six of these 8 responders described in a few keywords their guideline content or referred to a web-based guideline. CT with contrast enema per rectum, diagnostic laparoscopy, and endoscopy were the methods they described.

Core questions about reporting fluid collection in the pelvis, rectovaginal fistula and air, pus, or feces in drainage and watchful waiting as AL (questions 6 to 9) showed disagreement whether these events should be reported to DCCG as AL (Table 1).

In questions 10 to 13 the responders were asked to describe their perioperative procedures. All of the responders used a leak test with air insufflation. Ten of the responders used a rigid scope and 3 used a flexible scope.

In questions 14 to 20 the responders were asked to describe if they postoperatively used routine laboratory measurements or clinical algorithms for postoperative surveillance (Table 2 and Box 1).

The very last question was if the responders always performed a laparoscopy or a laparotomy if they had a confirmed AL. Two of the thirteen responded yes.

## 4. Discussion

The study shows that the reporting of AL to DCCG is not rigorous and unequivocal, and therefore the results of that specific parameter in the database should be interpreted with caution.

The response rate on 82% was high compared to most other studies. A systematic review of Internet-based surveys of health professionals found response rates, which ranged from 9% to 94% [5]. The high response rate in the present study could be explained by an e-mail reminder after 2 months followed by information on the project by phone [6–8].

In comparison a survey using the same online service was conducted among colorectal surgeons in UK. As in our study the objective was the definition of AL. A response rate on only 28.4% was achieved [9]. In that study extravasation of contrast on enema and fecal matter seen in pelvic drain or from the wound was accepted as diagnostic for AL.

To elucidate the validity of the questionnaire, ideally the results should have been compared to the patients records and case forms reported to the database for each separate department. Furthermore, it should have been compared to a national gold standard in the definition of AL. However in 2011 this was nonexisting but has been introduced from 2013 and onwards. The aim of this investigation was not to define the true incidence of AL but to investigate any differences in reporting AL to the DCCG database. None of the responders had any remarks concerning the understanding of the questionnaire. The validity of this questionnaire analysis therefore seems to be high.

Drainage of the small pelvis can be considered as indicator of AL [10]. Interestingly only half of the responders in this survey reported patients with air, pus, or feces in the drain as being an AL. Only half of the UK surgeons agreed that radiological collection treated with antibiotics or percutaneous drainage constituted an AL [9]. This is similar to the findings in our study, where only 38% reported a fluid collection in the small pelvis as an AL. It has been found that both patients with and without AL have fluid collection in the small pelvis [11]. Sixty-nine percent of the UK surgeons agreed that intra-abdominal sepsis requiring laparotomy constituted an AL. The precise formulation of their questionnaire was not apparent [9]. An increase in C reactive protein (CRP) may be a predictor of septic complications after elective colorectal surgery [12]. In our study 76.92% of the responders used daily measurements of CRP concentration on routine, and 31% routinely used postoperative clinical scoring systems or algorithms. The use of the postoperative surveillance programs may detect more subclinical AL resulting in a higher frequency of reported AL in these departments.

The international study group of rectal cancer (ISREC) [13] has proposed a definition for AL and suggested grading system for AL according to clinical severity: firstly the AL should be defined as a defect of the intestinal wall integrity at the colorectal or coloanal anastomotic site (including suture and staple lines of neorectal reservoirs) leading to a communication between the intra- and extraluminal compartments. A pelvic abscess close to the anastomosis should also be considered as anastomotic leakage. Grade A is an AL requiring no active therapeutic intervention, grade B is an AL requiring active therapeutic intervention but manageable without relaparotomy, and grade C is an AL requiring relaparotomy.

ISREC validated the definition and severity grading in a cohort of 746 patients [14]. ISREC concluded that their definition and grading system of AL may facilitate comparisons of results from different studies on AL after sphincter-preserving rectal surgery. Only 16% of the patients had a grade A AL. In a recent study from 2014, which included 129 patients with low anterior resection, the ISREC definition and severity-grading system was applied. Of 19 patients with contrast enema proven AL, 61% had grade A, 17% grade B, and 22% grade C [15]. These results show that this new grading system has its own shortcomings. The only way to get precise information on the true incidence and possible consequences for the patient is a routine CT scanning with contras enema at a fixed and generally accepted postoperative day. This approach will also elucidate those AL, which are hidden by a diverting stoma.

After this study was presented in Danish as an abstract to the Danish Surgical Society's annual meeting 2013 changes have been made.

The Danish surgeons are now asked to report if the AL do not demand treatment, demand treatment but not surgery, or demand relaparotomy or relaparoscopy and if the anastomosis is taken down.

## 5. Conclusion

There is a demand of more precise knowledge on the rate of AL and the possible consequences on disease-free survival, morbidity, and functional outcome. We suggest a multicenter prospective study, where the proposed ISREC definition and severity grading of AL are combined with a CT scan with rectal administration of contrast and measurement at a fixed postoperative day.

## Figures and Tables

**Box 1:**
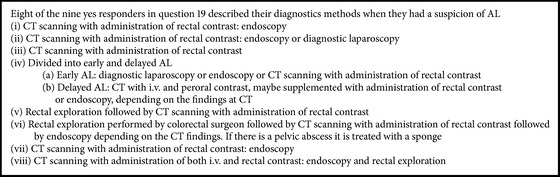


**Table 1:** Core question about fluid collection, rectovaginal fistula, drainage, and watchful waiting.

Question		Yes		No		Always		Some times		Never		
		*n*	%	*n*	%	*n*	%	*n*	%	*n*	%	
6	Do you report patients to DCCG with a fluid collection in the small pelvis as a leakage - regardless of the patients has a radiologic or endoscopic proven leakage?	5	39	8	61							

7	Do you report patients to DCCG with rectovaginal fistula as a leakage?	8	62	5	38							

8	Do you use drainage close to the anastomosis?					4	31	5	38	4	31	If always or sometimes the responder was asked to answer question 9

9	Do you report patients with air, pus or faeces in the drain as a leakage if no leakage is shown by reoperation, radiology or endoscopy?	5	56	4	44							

22	Do you occasionally use watchful waiting in patients suspicious of anastomotic leakage?	7	54	6	46							If yes the responder was asked to answer question 23

23	Do you report watchful waiting patients to the DCCG database as a leakage?	6	86	1	14							

**Table 2:** Questions about postoperatively used routine measurements or algorithms.

		Yes		No		
		*n*	%	*n*	%	
14	Do you postoperative on routine and daily basis measure C-reactive protein [CRP)?	10	77	3	23	

15	Do you postoperative on routine- and daily basis measure other biomarkers such as D-dimer, procalcitonin, cytokines or others?	1	8	12	92	The one yes responder measured cytokines as part of a project.

16	Do you postoperative on routine basis use clinical scoring systems or algorithms?	4	31	9	69	If yes the responder was asked to answer question 17

17	Kindly describe the clinical scoring systems or algorithms you use					Four of the responders described their clinical scoring systems or algorithms. They were based on “early warning system” EWS

18	Do you always use the same diagnostics methods in the same order when you have a suspicion of AL?	9	69	4	31	If yes the responder was asked to answer question 19 and If no the responder was asked to answer question 21

19	Kindly describe the diagnostics methods in the same order?	The answer is shown in Box 1				

20	Kindly describe the diagnostics methods in different order?					The 4 responders answered that they on suspicion of AL used different diagnostics methods in different order. Three of the four responders described that the choice of method and order depended on the patients clinical condition and the surgical approach (open versus laparoscopic).
